# Temporal resolution trumps spectral resolution in UAV-based monitoring of cereal senescence dynamics

**DOI:** 10.1186/s13007-024-01308-x

**Published:** 2024-12-19

**Authors:** Flavian Tschurr, Lukas Roth, Nicola Storni, Olivia Zumsteg, Achim Walter, Jonas Anderegg

**Affiliations:** 1 Department of Environmental System Sciences, Institute of Agricultural Sciences, ETH, Zurich, Switzerland; 2 Department of Environmental System Sciences, Institute of Integrative Biology, ETH, Zurich, Switzerland

**Keywords:** High throughput field phenotyping, Wheat, Cereal, Senescence dynamics, UAV, Spectral indices

## Abstract

**Background:**

Senescence is a complex developmental process that is regulated by a multitude of environmental, genetic, and physiological factors. Optimizing the timing and dynamics of this process has the potential to significantly impact crop adaptation to future climates and for maintaining grain yield and quality, particularly under terminal stress. Accurately capturing the dynamics of senescence and isolating the genetic variance component requires frequent assessment as well as intense field testing. Here, we evaluated and compared the potential of temporally dense drone-based RGB- and multispectral image sequences for this purpose. Regular measurements were made throughout the grain filling phase for more than 600 winter wheat genotypes across three experiments in a high-yielding environment of temperate Europe. At the plot level, multispectral and RGB indices were extracted, and time series were modelled using different parametric and semi-parametric models. The capability of these approaches to track senescence was evaluated based on estimated model parameters, with corresponding parameters derived from repeated visual scorings as a reference. This approach represents the need for remote-sensing based proxies that capture the entire process, from the onset to the conclusion of senescence, as well as the rate of the progression.

**Results:**

Our results indicated the efficacy of both RGB and multispectral reflectance indices in monitoring senescence dynamics and accurately identifying key temporal parameters characterizing this phase, comparable to more sophisticated proximal sensing techniques that offer limited throughput. Correlation coefficients of up to 0.8 were observed between multispectral (*NDVIred668*-index) and visual scoring, respectively 0.9 between RGB (*ExGR*-index) and visual scoring. Sub-sampling of measurement events demonstrated that the timing and frequency of measurements were highly influential, arguably even more than the choice of sensor.

**Conclusions:**

Remote-sensing based proxies derived from both RGB and multispectral sensors can capture the senescence process accurately. The sub-sampling emphasized the importance of timely and frequent assessments, but also highlighted the need for robust methods that enable such frequent assessments to be made under variable environmental conditions. The proposed measurement and data processing strategies can improve the measurement and understanding of senescence dynamics, facilitating adaptive crop breeding strategies in the context of climate change.

**Supplementary Information:**

The online version contains supplementary material available at 10.1186/s13007-024-01308-x.

## Introduction

A significant portion of daily calorie and protein intake is based on a handful of arable crops, notably wheat. Consequently, there is an immediate need to identify and mitigate risks associated with the cultivation of these crops [1]. This urgency is further accentuated by the projected decline in global wheat production and quality, including protein content and composition, due to the impacts of global climate change [2, 3]. Potential mitigation strategies encompass diversifying cropping systems or developing crop varieties with enhanced performance under stress conditions [4]. An important stress-adaptive trait that is effective in several crops is the prolonged maintenance of green leaf area after anthesis to increase carbon assimilation during grain filling [5]. In wheat, the primary limiting factor for potential grain yield is seen predominantly in sink strength, which refers to the number of grains available for grain filling and their capacity to absorb assimilates. This critical determinant is largely established before grain filling initiates, extending up to and including a short period after anthesis, as extensively reviewed by Borrás et al. [6], Fischer[7] and Distelfeld et al. [8]. Nevertheless, several studies have documented a positive correlation between delayed senescence and grain yield, especially in combination with stress conditions [9–13]. In addition, the onset of senescence marks a basic transition of canopies from carbon assimilation to N remobilization [14], and its timing is therefore expected to affect not only grain yield but also grain protein concentration, which is a key quality parameter of major economic importance. Though positive effects of a delayed senescence on yield are frequently observed, adverse effects on yield, harvest index, grain protein content, and remobilization efficiency have also been reported occasionally [15–17].

In situations in which severe stress challenges wheat, a prolonged retention of green leaf area may be interpreted as the prevention of premature senescence. An example of this is the CIMMYT wheat line SeriM82, which has a significant stay-green related yield advantage in Australian multi-environment trials over locally adapted genotypes [18]. In this case, a denser root system at depth likely increases deep soil moisture extraction late in the season, supporting an extended grain filling duration by delaying stress-induced senescence, resulting in increased individual grain weight [18–21].

Understanding this yield-determining phase in wheat is critical, especially in light of future climate change and more extreme events [22]. Even in countries with temperate conditions, such as Switzerland [23], a change in the phenology pattern [24] and an increase in agriculturally relevant climate extremes, such as heat waves, is expected [25].

Traditionally, visual greenness scorings are used to track senescence in breeding. More technical, sensor-based approaches such as SPAD meter measurements at the leaf level and green-seekers, Red-Green-Blue (RGB) images, or hyperspectral devices at canopy level were evaluated as tools as well [10, 13, 19, 26–29]. Compared to visual scorings, sensor-based approaches offer the potential to reduce scorer bias, thereby enhancing the reproducibility of assessments. Furthermore, they can support the automation of the data acquisition process, thus providing the required throughput. Depending on the sensors used, more or less detailed information can be retrieved from the measurement (e.g., detailed biochemical and physiological information), at least in principle e.g., [30–32]. However, sensors such as the SPAD meter or color measurements on the leaf itself sample only a small part of the leaf. As senescence typically does not uniformly progress on the leaf, additional problems occur to obtain robust values for a whole plot or canopy [13].

The study of senescence dynamics is often based on temporally sparse assessments of the senescence status, representing snapshots in time. This has enabled the detection of major differences between genotypes in terms of late-season phenology and has even allowed for the mapping of related quantitative trait loci (QTL) [33]. However, temporally sparse measurements, even if optimally timed, do not support a detailed assessment of the dynamics of the senescence process. Furthermore, absolute values of RGB or multispectral indices at specific points in time can be significantly affected by other canopy-related traits, such as vegetation cover and canopy 3D-structure. This issue can be addressed by restricting the analysis to relative temporal changes at the plot level in time series of measurements [13, 34].

Increasing the throughput of measurements is critical for the assessment of complex dynamic traits due to the need for intense field testing and frequent in-season reassessment. A high measurement frequency can be achieved by the use of high throughput field phenotyping (HTFP) technologies, such as unmanned aerial vehicle unmanned aerial vehicle (UAV)s. Several studies have evaluated the potential of UAVs equipped with RGB or multispectral sensors to infer the senescence status of crop canopies [35, 36]. However, detailed assessments of the potential of such sensors deployed on UAVs to integrally track senescence as a dynamic process are lacking.

**Fig. 1:**
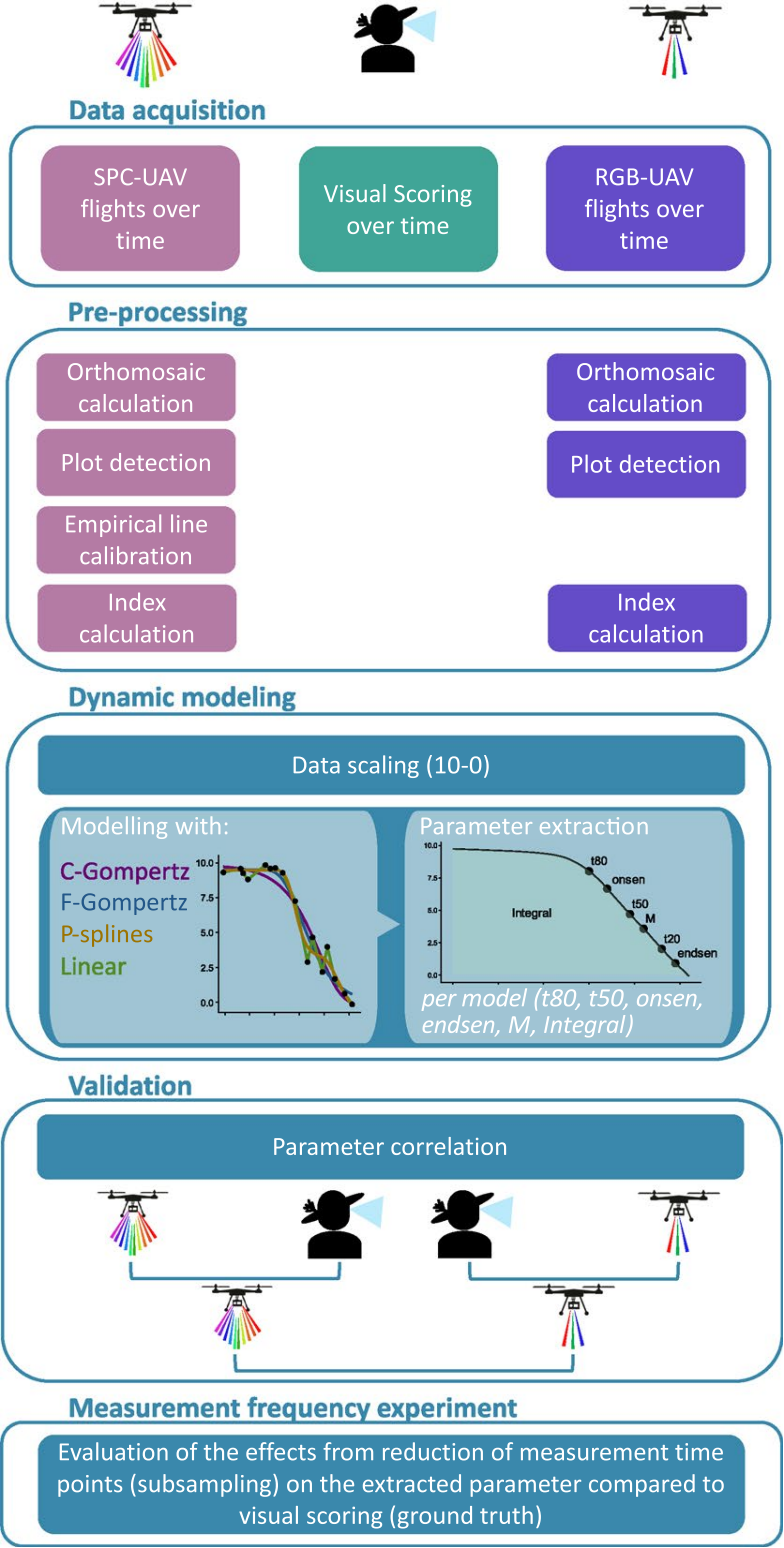
Overview of the workflow consisting of five main steps: (i) data acquisition using three different methods: ground truth visual scoring, multispectral (SPC) UAV measurements and RGB UAV measurements, all performed multiple times during the senescence phase; (ii) data pre-processing to combine individual UAV images into georeferenced orthomosaics on which individual plots are detected. The SPC images are additionally calibrated with the empirical line methods before the indices are calculated; (iii) different dynamic modeling approaches are tested (C-Gompertz, F-Gompertz, P-splines and Linear) from which key parameters describing the dynamics of senescence are extracted (*t*80, *onsen*, *t*50, *M*, *t*20, *endsen*, *Integral*); (iv) validation of parameters by selecting the best index and model for each sensor and correlating SPC and RGB with the visual scoring and with each other; (v) assessment of the influence of measurement frequency based on results from the preceding steps

The aim of this study was to (i) assess the potential of RGB and multispectral (SPC) indices extracted from UAV-based aerial imagery to track visually observed senescence as a dynamic process and (ii) to evaluate the relative importance of a high spectral and a high temporal resolution. This analysis provides important insights regarding the optimal allocation of resources for senescence monitoring in research and breeding programs. We conducted three winter wheat trials in two years involving 645 phenologically and morphologically diverse winter wheat genotypes, which provided a broad basis for method calibration and validation. Traditional visual assessments and UAV-based aerial imaging of the experiments were conducted in parallel and used to gauge sensor performance against visual scoring. Data set sub-sampling in combination with dynamic modelling yielded insights into the relevance of a high temporal and spectral resolution (Fig. 1).

## Materials and methods

### Field experiments and data

**Fig. 2:**
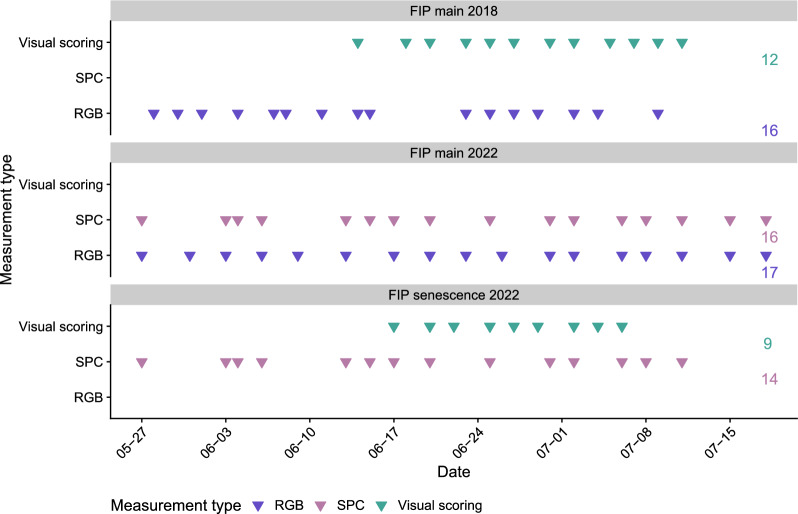
Each panel shows one of the three experiments that were conducted. The *FIP main* in 2018 is described in more detail in Anderegg et al. [13] and the *FIP senescence* experiment in 2022 in Anderegg et al. [40]. The x-axis shows the date measurements were obtained: visual scoring, RGB, or multispectral (SPC). The numbers at the end of the column show the total number of measurement campaigns for each measurement type per experiment

Field experiments were conducted at the ETH Zürich research station for Plant Sciences Lindau-Eschikon, Switzerland (47.449 N, 8.682E, 520 m a.s.l.) within or right next to the Field Phenotyping Platform (FIP) [37]. The experimental field was a gleyic cambisol soil with 21% clay, 21% silt and 3.5% organic matter [37]. Data originating from three different experiments were used. The experiments were carried out in 2018 and 2022 which represented two dry (especially 2018) and warm (especially 2022) years (Fig. 2; see Figure A1 showing precipitation and temperature during the experimental years). Each of the RGB, SPC, and visual assessments of senescence were obtained in two out of the three experiments (Fig. 2). The FIP main experiments were replicated twice, with each replication consisting of 396 experimental plots. The FIP senescence experiment consisted of 180 experimental plots. Experiments were sown at the end of October each year, and crop husbandry was performed in accordance with the local agricultural practices. A broad genetic diversity was reached in these experiments by growing the GABI wheat panel, more than 300 breeding lines, [38, 39] as well as various additional European and Swiss cultivars on overall 2160 experimental plots. The measurements started around the growth stage of heading. On average, measurements were conducted every 2 to 7 days (Fig. 2), although the frequency of measurements was subject to environmental conditions, such as rainfall.

#### Visual scoring

Reference assessments of senescence were obtained through visual inspection of canopies at regular intervals, starting before the onset of senescence in the earliest-maturing genotype and continuing until the latest-maturing genotype reached maturity. Canopies were inspected at a view angle of approximately $45^{\times }$, lower leaf layers were manually exposed where necessary to enable inspection. All estimates were made for a central region of about 0.5 m × 0.5 m in each plot. In 2019, canopy senescence was assessed by estimating the fraction of green, chlorotic, and necrotic leaf and stem area, with chlorotic areas interpreted as representing an intermediate stage of senescence. The resulting average impression of the advancement of senescence was expressed as an integer value on a scale ranging from 0 (representing complete canopy senescence) to 10 (representing fully stay-green canopies). Ear senescence was not considered. In 2022, the focus of the visual scorings was put on the remaining green leaf area, whereas stem and ear senescence was not considered, and no distinction was made between chlorotic and necrotic tissue, with both considered as “senescent”. Example images with the corresponding visual scorings are provided in Figure A2. All visual scorings were made by a single person to avoid scorer-bias. Data was recorded using the field book app [41].

#### Sensors

For RGB image acquisition (Fig. 1 violet), a camera with a full frame sensor of 6000 × 4000 pixel (Sony $\alpha$ 9 Model ILCE-9, Sony Corporation, Tokyo, Japan) equipped with a prime lens with a focal length of 55 mm and a maximum aperture of f/1,8 (Sonnar T $*$ FE 55 mm F1,8 ZA, Sony Corporation, Tokyo, Japan) was used as described in Roth et al. [42]. For the SPC image acquisition (Fig. 1 rose) a 10 band multispectral sensor (Micasense Red Edge MX dual cam system, MicaSense, Seattle, United States) was used (see Table A1). Both sensors were mounted to a Matrice 600 Pro drone (SZ DJI Technology Co. Ltd., Shenzhen, China) and attached to a Ronin-MX gimbal (SZ DJI Technology Co. Ltd., Shenzhen, China) in order to minimize measurements with off-nadir position. The used multispectral and RGB sensors currently cost approximately 12000 and 1500 USD, respectively.

#### UAV measurements

The RGB-UAV flights were performed at 28 m altitude with front overlap ratio of 90 % and a side overlap ratio of 80 %. This resulted in a ground sampling distance (GSD) of 3 mm. The experimental field was equipped with ground control point (GCP)s arranged cross-wise with a spacing or 12 m × 18 m to enable precise georeferencing, as described previously [42]. The SPC campaigns were conducted according to Perich et al. [43] with a flight height adjusted to 100 m. The resulting GSD for the SPC images was $\sim$ 5 cm. We aimed for a minimum of two measurements per week. However, measurements with the multispectral sensor require specific measurement conditions (very stable light conditions in particular). Therefore, maintaining a rigid measurement schedule was not always possible. The objective was to conduct the measurements with the different sensors on the same day and at approximately the same time. In general, the measurements were carried out as close as possible to the maximum solar elevation, in the early afternoon with as stable a light conditions as possible.

### Image (pre-)processing

RGB and SPC images were pre-processed in a similar pipeline using Agisoft PhotoScan Professional 1.4.2 and Agisoft Metashape 1.5.2 (Agisoft LLC, St. Petersburg, Russia) and OpenCV [44]. Images from a given flight were first aligned, and camera position estimations referenced using GCPs placed in the field. The world-coordinate positions of the GCPs were measured with a Trimble GPS RTK (R10, Trimble Ltd., Sunnyvale, U.S.A.) with a maximal error of 2 cm. From a generated dense point cloud, a digital elevation map was extracted and orthophotos were projected onto it. This orthophoto was used only to define the region-of-interest (ROI) of plots, but not for data extraction. To avoid fusing pixels from different shots and height-related artifacts, these ROIs were back-projected to the original images, according to Roth et al. [42]. Plot-values were then extracted from the most nadir-view image per plot.

In order to minimize the impact of fluctuating environmental conditions, such as variations in lighting across individual flights, a calibration was conducted for the SPC measurements using reference panels with known reflectance(0 %, 15 %, 25 %, 75 % and 100 % black). Their reflectance’s were used to fit a linear model according to the empirical line method [45], and predict the calibrated multispectral values. Calibration of the SPC images was done after the clipping of individual plots from orthophotos.

Vegetation indices (see Tables A2 and A3 based on [46–59]) were calculated based on reflectance or RGB values of a central region of interest within each plot. A margin of approximately one sowing row (12.5 cm) on each side of the plot was maintained to reduce border effects and the median value of all pixels was exported for further analyses. This area also corresponds to where the visual scoring values were obtained in the experimental plots. 25 and 16 indices were extracted from the SPC and the RGB imagery, respectively.

### Dynamic modelling

Various approaches for modelling the dynamics of senescence based on repeated visual scoring, measurements of green leaf area, or spectral indices were proposed, and different sets of parameters are often extracted from model fits [60]. The choice of model and parameters depends on the level of assessment and on the frequency with which these assessments are made. At the canopy level, senescence typically shows a sigmoidal progression, which can be well represented by two-parameter or four-parameter logistic-type models. Alternatively, splines may provide more flexibility to represent atypical senescence curves as well, or may be used to model spectral indices that deviate in their dynamics from visual observations. However, using more flexible approaches increases the risk of overfitting to errors associated with single measurements or scorings when compared to more rigid fully parametric approaches. Here, we evaluated both approaches. Given that our main interest is in extracting the dynamics of senescence rather than absolute values at a specific point in time, all scorings and measurements were scaled at the plot level to range from 0 to 10, representing the minimum and maximum value recorded for the assessment period, respectively. Four-parameter Gompertz models were fitted as described earlier (F-Gompertz; *fgom*) [13]:

1 $$\begin{aligned} S =A + Ce^{-e^{-b\times (t-M)}} \end{aligned}$$

where *S* represents the trait value; *A* and *C* are the lower and upper asymptotes, respectively; *b* is the rate of change at time *M*; and *M* is the time point when the rate is at its maximum [61]. A corresponding two-parameter model with asymptotes constrained to 0 and 10 was also fitted (C-Gompertz; *cgom*). Monotonically decreasing P-splines (*pspl*) were fitted using the R-package scam [62], with the number of spline knots set to three quarters of the number of observations, as done for similar data by Roth et al. [63]. The fourth used approach was a linear (*lin*) interpolation between the data points. To simplify modelling and model interpretation, the scale for indices with increasing values during senescence was inverted. The different models were applied to the visual scoring as well as to all indices. The correlation between visual scoring and the HTFP method, using the equivalent model for each, was then calculated (see Fig. 1).

### Extracting key time points

**Fig. 3:**
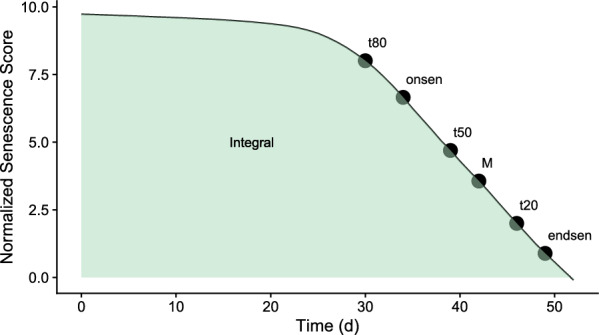
The seven extracted parameters are plotted on an index trajectory. The x-axis represents time in days after the first measurement, the y-axis represents the normalized senescence score value). Six of the parameters are time points (*t*80, *onsen*, *t*50, *M*, *t*20 and *endsen*), and one describes the area below the index curve in green (*Integral*)

From the fitted models, a value was predicted for each plot on each day in the assessment period. The predicted values were used to extract the parameters *t*20, *t*50 and *t*80 as the time points when modelled scaled scorings or index values decreased to 8, 5, and 2, i.e., when values decreased to 80 percent, 50 percent, and 20 percent of the maximum observed value within the assessment period. Additionally, the *Integral* under the fitted curves was extracted. For fully parametric models, we also extracted the *M* parameter (see above - the inflection point) directly from the model fit. Finally, the onset (*onsen*) and endpoint (*endsen*) of senescence were extracted from parametric models as the time point when the second derivative was at its minimum and maximum, respectively (Fig. 3). These points were chosen because the second derivative mathematically describes the acceleration of a process. Therefore, minima and maxima of the second derivative are theoretically defined to be closely related to the start or end of a logistically shaped process. Methods for dynamic modeling and key parameter extraction can be found in the public open git repository.

### Best index selection

The best index-model combination was selected based on dynamic modeling and derived key time points. This approach reflects the idea that the suitability of a sensor-based proxy for senescence should be judged based on its capability of capturing senescence as a dynamic process. As a robust value for the overall goodness of an index the median correlation coefficient across all extracted parameters for each index was computed. This was done by calculating the correlation coefficient for each parameter, without reference to the underlying model, and then computing the median value over all derived correlation coefficients. The median was selected to prevent the indices from being negatively biased by low or high outliers of correlation coefficient values derived from single index-model combinations. In addition, the sum of all correlation coefficients as well as their range was extracted.

Selection of the optimal model was based on the selected index, using the same metrics. Note that this approach does not take into account any absolute temporal shift or bias of an index. We checked manually if such a bias was present and adapted the selection accordingly if meaningful. The sensors are compared using the extracted parameters and their correlation between the best selected index and visual scorings. Additionally, we directly compared the two sensors against each other based on the parameters *t*20, *t*50 and *t*80. For each of the parameters, the best index-model combination of each sensor was taken and the Pearson correlation as well as the root mean squared error (RMSE) was calculated across all measured plots.

### Temporal measurement density effects

To evaluate the effect of measurement frequency on the estimation of senescence dynamics parameters, we generated subsets of the full data set that contained a decreasing number of measurement time points (n). Nine independent subsets were sampled per n, with n = 5, 6,..., 16. This procedure was repeated twice: Once enforcing the first and last measurement time point to be contained (“fixed”) in the subset, and once relaxing this constraint (“free”). Each resulting subset was then subject to dynamic modelling and parameter extraction as described above. The analysis was performed for the best index-model combination identified previously on the measurements of the FIP main and senescence experiments in 2022, for which also visual scorings were available. Correlation coefficients between the visual scoring and HTFP derived parameters were computed in a subsequent step.

## Results

Visual scorings were used as a reference for identifying the optimal index and model for subsequent evaluation of both the sensor.

### Best index and model selection

**Fig. 4:**
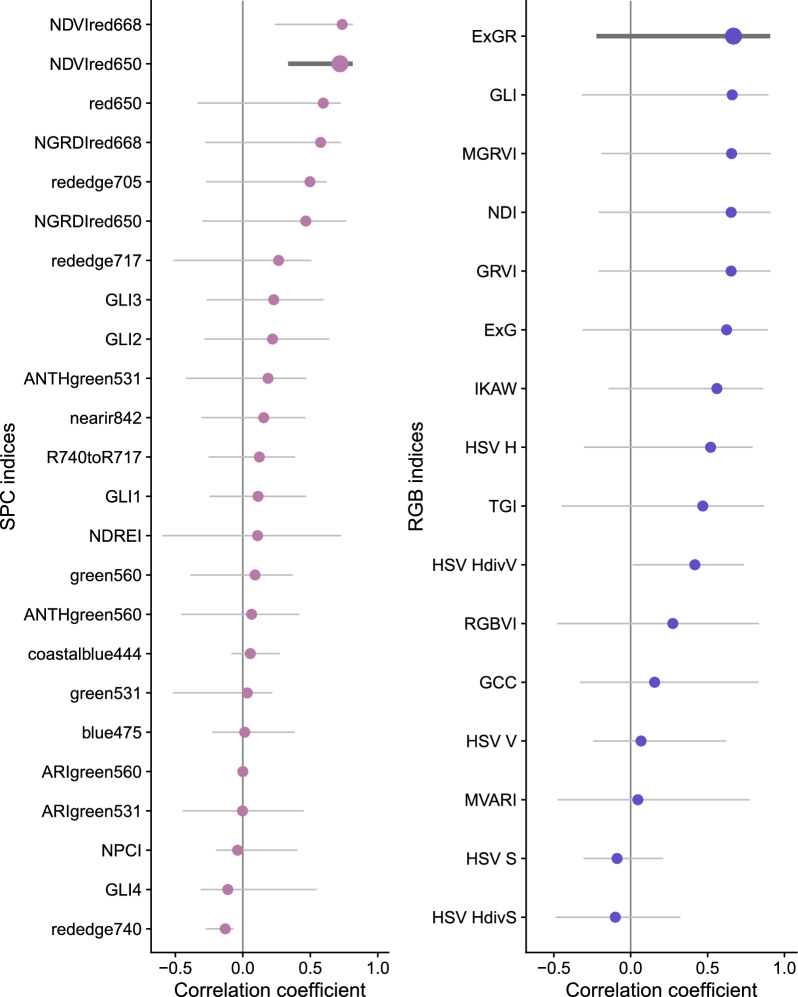
The selection of the best index for SPC (left) and RGB (right) was done according to the median (points) and the range (minimal and maximal - whiskers) of the correlation coefficient values over all used parameters between the parameter extracted from the corresponding sensor and the visual scoring. The thicker line represents the selected best index (*NDVIred*650 and *ExGR* for SPC and RGB respectively)

**Fig. 5:**
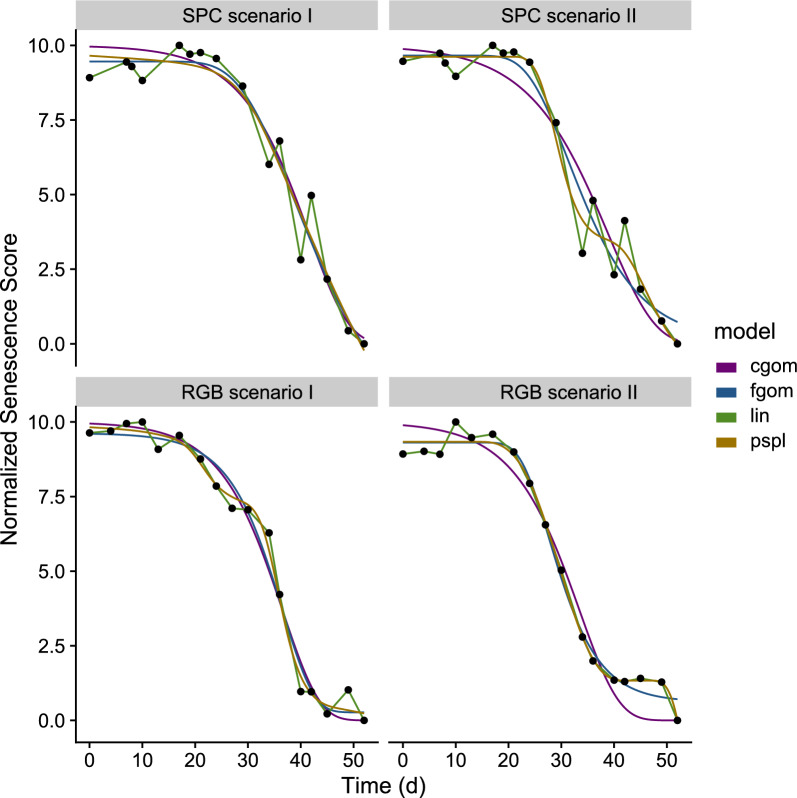
For two experimental plots the different model fits are shown (lines), in the upper row for the selected SPC index (*NDVIred*650) in the lower row for the selected RGB index (*ExGR*). The points represent the scaled measurement index values. *ScenarioI* represents plots with stable data where there are no large differences between the model fits. *ScenarioII* represents plots with unstable and noisy data, where there are differences between the model fits and hence parameter extraction

For visual scorings, the type of model applied had a negligible effect on the estimation of the senescence dynamics parameters (all pairwise correlations > 0.97; Figure A11 and Table A4). According to the overall correlation of parameters with visual scorings, for SPC the *NDVIred*650 and for RGB the *ExGR* revealed as the best indices (Fig. 4). For the SPC indices, *NDVIred*668 had a slightly higher median correlation (0.73 versus 0.72) but a wider range of correlation values (0.58 versus 0.48) across all parameters of interest than *NDVIred*650, hence the latter was preferred. *ExGR* was clearly the best RGB index in both aspects. Overall, the *cgom* (C-Gompertz) model provided the best approximation of the correlation range difference, and exhibited a comparable value to the *fgom* (F-Gompertz) model in terms of the correlation sum (Table A5). The *cgom* model appeared to be more meaningful than the *fgom* model in following the actual senescence dynamics: While the *fgom* has more degrees of freedom, it is more prone to following measurement errors (Fig. 5; right side). The same holds true for the *pspl* and *lin*. This issue arose particularly with noisy index trajectories or larger uncertainties between two measurements (Fig. 5; *scenarioI* versus *scenarioII* plots), otherwise, the different models yielded very similar results (see also Figures A3, A4, A5 and A6). In *scenarioI*, the data are considered to be very stable, enabling all of the specified models to represent the senescence dynamics with minimal discrepancies. In contrast, *scenarioII* comprises plots with noisy or unstable data. Consequently, the application of different modelling approaches will also result in disparate model fits, thereby introducing discrepancies in extracted parameters.

### Validation

**Fig. 6:**
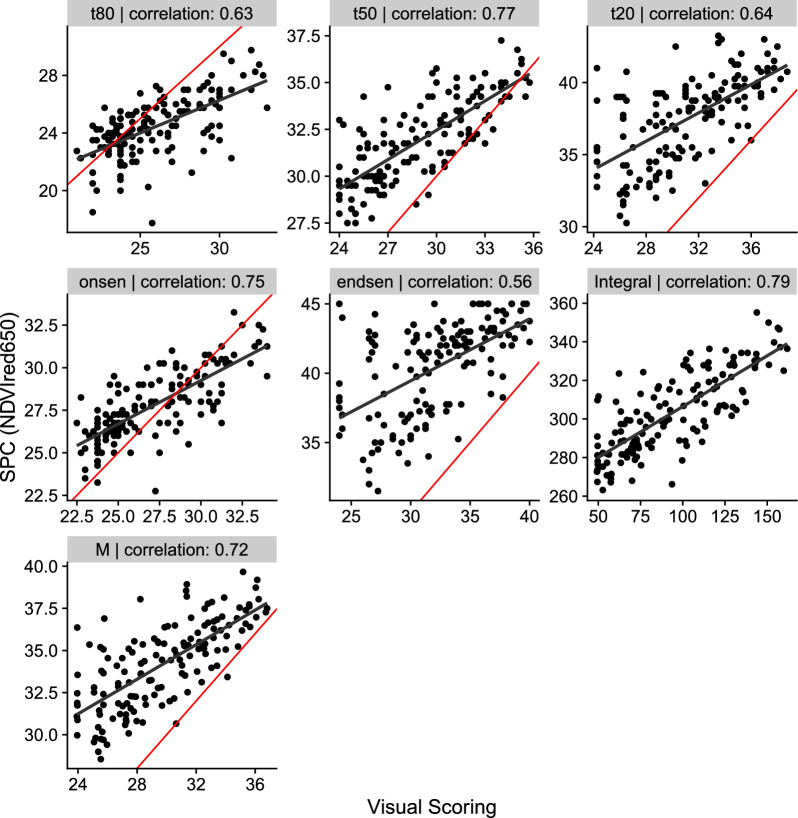
Correlation between the senescence dynamics parameters obtained from visual scoring (x-axis) and the best SPC index (*NDVIred*650 with the *cgom* model) measured by high throughput field phenotyping (HTFP). The red line represents the 1:1 line, while the black line shows the correlation. Each panel shows one parameter (indicated in the header) and the corresponding Pearson correlation value

The correlations between parameters estimated with visual scoring and drone-based methods was found to be strongest when using the *NDVIred*650-index with the *cgom* model for the parameters *t*80, *t*50, *onsen*, *Integral*, and *M* (Fig. 6). For the parameter *Integral* a moderate correlation but a substantial bias was observed. When considering the best index-model combination for each parameter individually, correlation coefficients ranged from from 0.73 (*t*80) to 0.82 (*t*80) (Figure A7). For five of the seven parameters the *NDVIred*650 or *NDVIred*668 were the best indices. However, just in two instances (*Integral* and *onsen*) the *cgom* model demonstrated superior performance compared to the *fgom* (*t*20, *endsen* and *M*) and the *pspl* (*t*80 and *t*50) model.

**Fig. 7:**
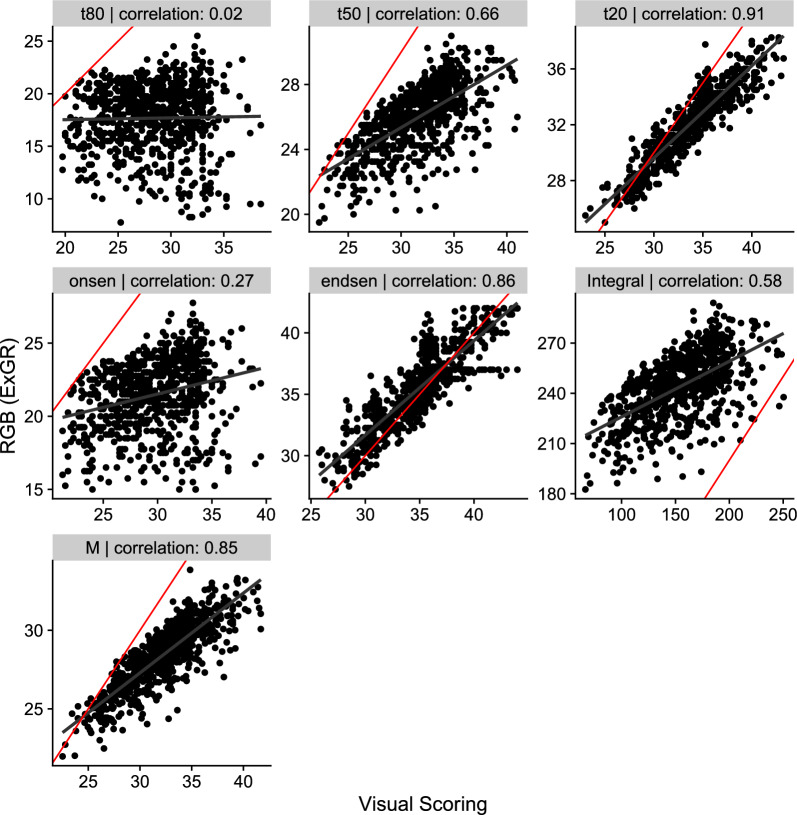
Correlation between the senescence dynamics parameters obtained from visual scoring (x-axis) and the best RGB index (*ExGR* with the *cgom* model) measured by high throughput field phenotyping (HTFP) (y-axis). The red line represent the 1:1 line, whereas the black line shows the correlation fit. Each panel shows one parameter (indicated in the header) and the corresponding Pearson correlation value

For the RGB indices, the *ExGR* with the *cgom* model showed overall the best performance (Fig. 4 and Table A5). Again, most of the parameters showed a strong correlation between RGB indices and visual scoring (*t*50, *t*20, *endsen*, *Integral*, and *M*), while *t*80 and *onsen* do not show a strong or any correlation (Fig. 7). By selecting the best combination for each parameter, we found correlation coefficients ranging from 0.32 (*t*80) to 0.91 (*t*20) (Figure A8). The *ExGR* index was found twice to be the best index per parameter as well (*Integral* and *M*), however, only once in combination with the *cgom* model.

A direct comparison between the two sensors via the dynamics parameters derived from the best RGB and SPC indices revealed correlation coefficients ranging from 0.54 (*endsen*) to 0.73 (*M*) in 2022 (Table A6 and Figure A9). Zero values occurred when a model converged but predicted values never crossed the pre-defined thresholds required for parameter extraction. This can for example occur in case of very noisy time series (e.g. for *onsen* a value of 8; see Figure A6). The resulting correlation for the year 2022 with all the parameters as well as RMSE values are shown in Table A6.

**Fig. 8:**
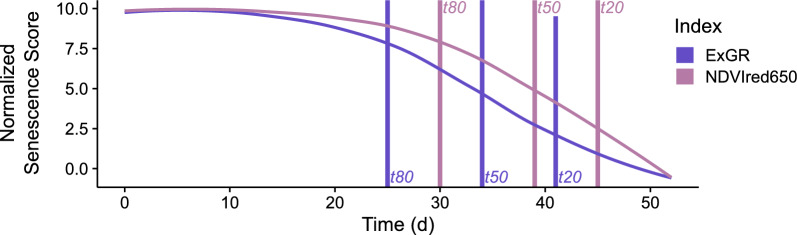
Senescence dynamics as measured via *ExGR* (RGB) *NDVIred*650 and (SPC) (y-axis median of the standardised senescence score of all measured plots in the FIP main experiment in the year 2022) over time (y-axis), starting from the first day of measurement (0) until the last day (53). Vertical lines represent the median of parameter *t*80, *t*50 and *t*20 measured by the different sensors

Taken together, the extracted parameters explain and define the dynamics of the senescence process. Figure 8 depicts the median of the best index of SPC and RGB over time. It can be observed that both indices exhibit a very similar trend, which explains the comparable results and the correlation between the parameters derived from the two sensors. Nevertheless, it is evident that there are still distinct differences in the dynamic, which allows for the observation of differences in the results and the derived parameters.

### Temporal versus spectral resolution

**Fig. 9:**
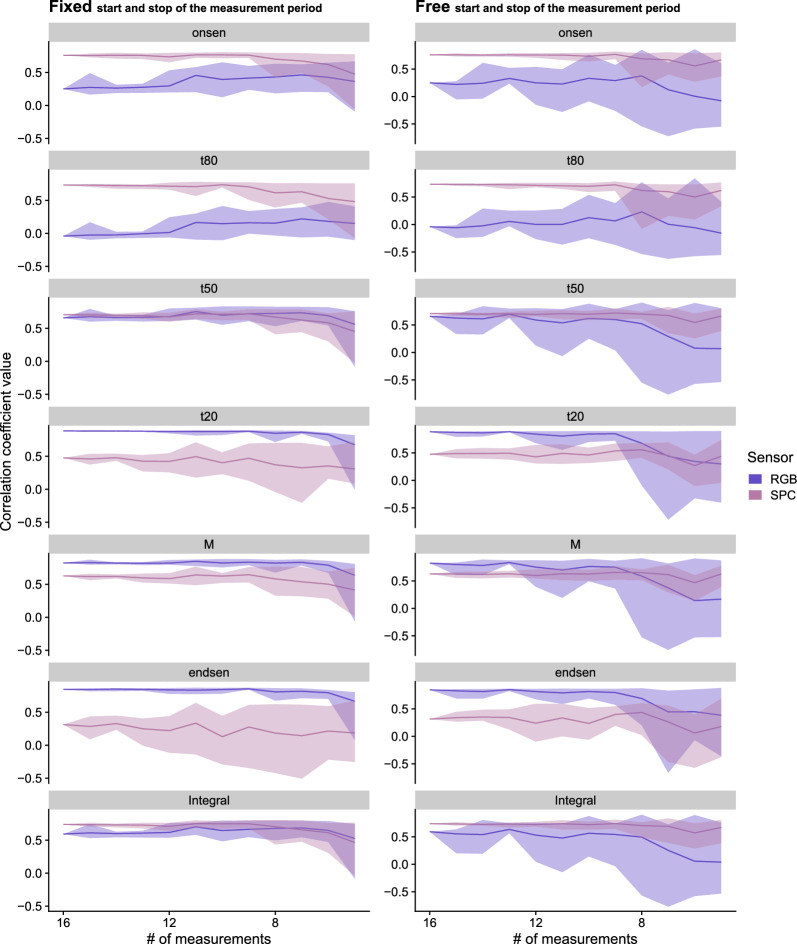
Correlation coefficients between visual scoring and HTFP derived parameters (y-axis) over the decreasing number of measurement time points (x-axis). Each row shows one of the parameters described above. The columns represent the different sub-sampling approaches, right with maintaining start and stop of the measurement period in the dataset, left without maintaining start and stop but freely reducing the number of measurement time points (from 16 to 5). The different colors indicate the two sensors (RGB - with *ExGR* and SPC with *NDVIred*650), the line represents the median, the shades the minimal and maximal values

Estimation of senescence dynamics parameters using sub-sampled time series generally resulted in decreased correlation coefficients (between visual scoring and HTFP) and larger spread of the correlation coefficient values in estimated parameters (Fig. 9). The overall tendency in both sampling strategies shows lower correlation coefficients and especially a wider spread of the values when reducing the measurement frequency. The left column of the Fig. 9 shows the sub-sampling results where the start and the stop of the measurement period were kept fixed in the dataset. In the right column the start and the stop were not constrained in the dataset, hence they may be in some subset but not necessarily (free start and stop of the measurement period). The correlation coefficients of the parameters at the very beginning or end of the senescence phase (e.g. *t*80 and *t*20) were less affected in terms of robustness and precision (i.e. bigger spread) than correlation coefficients of other parameters (e.g. *Integral*). The performance with fixed start and stop values was higher and more stable (lower range of of correlation coefficient spread) than with unconstrained sub-sampling. In particular, the SPC sensor showed a large increase in the range of the correlation coefficients and a reduction in correlation coefficient overall with a lower number of measurements.

## Discussion

We used a dataset containing three different experiments that were conducted over two years to evaluate the potential of UAV-based spectral and RGB imaging to capture the senescence dynamics in wheat. This comprehensive dataset represents more than 2000 individual plots and a large genotypic diversity, providing a vast basis for our analyses that may be applied to other, morphologically similar cereals as well.

For both sensors we identified a robust reflectance index that allows to track the senescence process, namely for the SPC the *NDVIred*650 and for the RGB sensor the *ExGR* index in combination with a dynamic model (*cgom*). By validating these sensors against visual scoring, the state of the art in plant breeding, we could show a strong correlation regarding several parameters (Figs. 6 and 7). We propose a robust method to extract key parameters of the senescence phase in a high throughput manner. Furthermore, our findings indicate that the accuracy of assessments is primarily determined by the temporal resolution of the measurements, rather than by the availability of narrow-band, specific spectral indices (Fig. 9).

### Potential of UAV-based senescence monitoring

The described method enables up-scaling of previously proposed approaches [10, 13, 19], allowing such methods to be implemented in breeding programs due to a feasible amount of work related to data acquisition. Furthermore, our results show that both evaluated sensors can yield comparable outcomes (Figure A9; Table A6) and provide a representation of the senescence process that is similarly accurate to that obtained by using more sophisticated sensors, such as spectroradiometers [13]. This indicates that cheaper RGB sensors can be used similarly, in line with results of Cao et al. [64]. Consequently, a notable reduction in cost is possible while comparable results can be maintained by selecting a less expensive RGB sensor over a SPC sensor (approximately 10 times cheaper). Also, such RGB sensors are widely used for other phenotyping purposes [42, 46, 65, 66]. Compared to SPC sensors, RGB sensors are more flexible regarding different illumination conditions, because settings for shutter speed or ISO can be readily adapted to the current situation.

However, this also implies that measurements can be made under less optimal conditions, which can facilitate more continuous monitoring of experimental plots. The results of this study confirm that the frequency and duration of measurement campaigns are more important than the chosen sensor. While a rough classification of genotypes into early or late maturing can be useful for many applications, several studies have highlighted the potential value of more detailed information regarding the dynamics of senescence. For instance, the rate or duration of senescence was found to correlate with remobilization efficiency, harvest index, grain protein concentration, and grain yield [15, 16, 29, 67–69].

### Effect of the temporal measurement density

Here, between 12 and 17 measurements were taken per experiment to track these dynamics. Previous studies used far fewer measurements (e.g., [33, 35]), which can introduce uncertainty. First, each measurement is associated with a certain error, as illustrated in Fig. 5 by the scatter between subsequent measurements. Although a reduction in the number of measurements will not increase the measurement error per se, it will give more weight to such errors, since there are fewer data points available to estimate the true trajectory. Second, diverse sets of genotypes are typically characterized by a continuous distribution in terms of phenology and the timing of senescence. Reducing the number of measurements will limit the degree to which minor differences between genotypes in this continuum can be resolved.

The temporal density of the measurements appeared to have a greater effect on the observed correlation between the HTFP and the scoring-derived parameters and their variance than the type of sensor used (Fig. 9). It is possible to extract certain parameters with comparable precision and variance even with a reduced number of measurement points (e.g. *t*80, *t*50, and *t*20) provided the beginning and end of the measurement-period are fixed at a physiologically meaningful point in time(Fig. 9, left panel). However, note that these results were obtained after extensive optimizations in terms of the index and dynamic model used for representation, relying heavily on temporally dense data. This prior knowledge may not be transferable across seasons and locations and would therefore have to be regenerated for each application scenario. We therefore argue that our results offer a very conservative estimate of the importance of measurement frequency. If the start and end of the measurement campaign are not fixed to meaningful points in time (Fig. 9 right column), the correlation coefficients show a higher variance with a reduced number of measurement time points. This trend can be observed for both sensors. Therefore, for a robust and meaningful parameter extraction and a better understanding of the observed period, continuous measurements over time as well as a meaningful timing regarding the start and end of the measurement campaign are essential. There are proposals to interpolate the measured features using an environmentally meaningful component (e.g. temperature) to fill measurement gaps [70]. However, this necessitates a vast quantity of data to train, which is unlikely to be available if the measurements were not made in a dense temporal resolution in the first place.

### Effect of the sensor choice

Nevertheless, the choice of sensor has an impact on the extracted parameters characterizing the senescence dynamics. When the measurement period was identical (as in FIP main 2022) both sensors were equal in accuracy, as evidenced by the high correlation coefficients (Table A6). When considering the best index per parameter for each sensor, higher correlation coefficients between visual scoring and HTFP derived parameters were observed (Figures A7 and A8). The indices exhibit a slightly different pattern over time (Figure A10). This may explain why the parameters can vary between the indices, and thus the correlation between the visual scoring and the HTFP derived measurement can be increased by selecting an index-model combination separately for each parameter. Such differences may be due to actual biophysical processes represented by specific indices. For example, one index uses band differences to represent water content of the canopy, while another index represents canopy greenness. The availability of additional bands in the SPC sensor allows for the identification of more potential combinations for indices, which may be of interest in the investigation of distinct senescence-related processes (e.g., chlorophyll degradation, changes in leaf area index, canopy desiccation, etc.). Tracking separate senescence-related processes can also facilitate disentangling the contribution of physiological senescence and stress to overall dynamics of green leaf area [34].

### Effect of the modelling approach

As illustrated in Fig. 5, there are also discrepancies between the distinct models utilized. The differences are minimal when the period is measured in high temporal resolution. In this case, the measurement error is also small (see also Figures A3, A4, A5, and A6). Conversely, if this is not the case, some models or indices follow a pattern that is not physiologically meaningful. Consequently, if one is interested in observing the full dynamic of a period and comparing it between different environments, we suggest using one index with one model, to be chosen as described in this study. This allows for a robust tracking of the phase of interest. Conversely, if the interest lies in extracting a single key time point, the precision can be maximized by selecting a specific index for each parameter. In our study, the selection of the best index-model combination per parameter increased the correlation between 0 and 0.48 for RGB and 0.01 to 0.19 for SPC (see Fig. 7 versus A8, respectively A7 versus A7). As shown in Fig. 4, the distinct models also exert an effect on the correlation score. While there is often little difference between the two types of Gompertz models, there is an overall tendency for both P-Splines and the linear model to perform worse. This suggests that fully parametric approaches are better suited to model the dynamics of canopy senescence.

### Combined effects and resulting considerations

The ability to measure at high temporal resolution allows for a more profound comprehension of senescence dynamics. These dynamics can then be employed to identify potential stressors, as exemplified by the approach taken in Tschurr et al. [71] for dynamic canopy cover measurements. In a subsequent step, this approach could be implemented in crop models to further improve prediction accuracy by accounting for growth stress according to measurable traits [72–75]. This approach could also be used to assess and enhance the performance and phenological adaptations of crop mixtures in a high-throughput manner [76].

The RGB sensor has a higher GSD than the SPC sensor, yet spatial resolution was insufficient to distinguish between plant and soil pixels or between different plant organs. These segmentation tasks require a very high spatial resolution, particularly during the senescence phase, because senescing or senescent plant material is easily confounded with soil background. This would require ground-based image acquisition Anderegg et al. [40]. Currently, ground-based, plot-by-plot imaging contradicts the need for high-throughput measurements required for routine assessment of senescence status across large germplasm collections in breeding programs. Here, the visual senescence scoring method was slightly different across years, including in the way in which chlorotic tissue and the contribution of different organs were weighted. These differences in the scoring method may have introduced a certain year-specific bias in the observed correlation between scorings and reflectance signatures. However, given that dynamics over time rather than absolute values at specific points in time were compared, this should not have falsified our results. Such issues could be elegantly solved in future work by using very high resolution imagery enabling the monitoring of organ-level senescence dynamics and an explicit distinction between chlorosis and necrosis [40]. This could allow for the disentanglement of flag leaf senescence, whole plant senescence, grain maturation and their corresponding genotypic patterns [60]. A reduction in the flight height of the UAV, in conjunction with an adapted flight strategy (i.e. [77]), which employs a high-resolution RGB camera, could facilitate the capture of the requisite data for this disentanglement. Nevertheless, the resolution of SPC sensors is typically lower, which suggests that further investigation and development of methods using RGB sensors is warranted.

## Conclusion

This study highlights the promising potential of scaling up and improving the efficiency of senescence measurements by using UAV. We proposed a strategy that prioritizes increased measurement frequency over time, as opposed to investing in more expensive sensors equipped with a high number of spectral bands. Ideally, a measurement campaign should be conducted every third day, depending on the weather conditions, or at least several times a week to yield robust results. Our investigation showed similar results when employing a standard RGB sensor as opposed to a 10-band SPC sensor, consistent with previous research using hyperspectral sensors. Our results highlighted the importance of dynamic temporal high-resolution measurement campaigns for traits such as senescence in winter wheat. Dynamic processes that exhibit substantial (G$\times$E) interactions require the use of dynamic modeling for a precise estimation of descriptive parameters. Large-scale analysis of germplasm using the proposed tools will contribute to the advancement of our understanding of the mechanisms affecting these dynamic traits and support the selection of well-adapted genotypes.

## Supplementary Information

Supplementary Material 1.

## Data Availability

Code and example data available at: dynamic_senescence_modeling.git.
