# Supplementary material for: Temporal resolution trumps spectral resolution in UAV-based monitoring of cereal senescence dynamics
Source: Plant Methods. 2024 Dec 19;20:188. doi: 10.1186/s13007-024-01308-x (PMC11657122; doi:10.1186/s13007-024-01308-x)
Supplement: Supplementary file 1 — Supplementary Material 1. [file 13007_2024_1308_MOESM1_ESM.pdf]

Appendix A Supplementary Information

A.1 Supplementary Tables

Table A1 Bandwidths of the Micasense Dual Camera system

Band Name	Center Wavelength (nm)	Bandwidth (nm)
blue475	475	32
green560	560	27
red668	668	14
rededge717	717	12
nearir842	842	57
coastalblue444	444	28
green531	531	14
red650	650	16
rededge705	705	10
rededge740	740	18

Table A2 The used RGB-indices are shown in the table below, whereas r refers to the red-color band, g to the green and b to the blue one. Furthermore the Hue, Saturation, Value color space (HSV) was used whereas the h stands for the hue value, s the saturation and v for the value. In the TGI following λ-values were used: $\lambda_r = 670$; $\lambda_b = 480$; $\lambda_g = 550$

Index	Formula	Reference
TGI	-0.5 × ((λ_r - λ_b) × (r - g) - (λ_r - λ_g) × (r - b))	used in [46]
ExG	$2 \times g - (r + b)$	used in[46]
NDI	$128 \times (\frac{g-r}{g+r} + 1)$	used in [46]
ExGR	$(2 \times g - (r + b)) - (1.3 \times r - g)$	used as in [46]
GCC	$\frac{g}{b} + g + r$	used in [47]
GRVI	$\frac{g-r}{g+r}$	used in [48]
IKAW	$\frac{r-b}{r+b}$	used in [49]
MGRVI	$\frac{g^2-r^2}{g^2+r^2}$	used in [50]
MVARI	$\frac{g-b}{g+r-b}$	used in [50]
RGBVI	$\frac{g^2-b\times r}{g^2+b\times r}$	used in [51]
HSV_H	h	
HSV_S	s	
HSV_V	v	
HSV_HdivS	$\frac{h}{s}$	
HSV_HdivV	$\frac{h}{v}$	

Table A3 Overview of the applied multispectral indices in this study. The first 10 rows are just each single band from the Micasense sensor.

**Index**	**Formula**	**Reference**
blue475	$blue475$	
coastalblue444	$coastalblue444$	
green531	$green531$	
green560	$green560$	
nearir842	$nearir842$	
red650	$red650$	
red668	$red668$	
rededge705	$rededge705$	
rededge717	$rededge717$	
rededge740	$rededge740$	
ANTHgreen531	$rededge740 \times \left(\frac{1}{green531} - \frac{1}{rededge705}\right)$	[52]
ANTHgreen560	$rededge740 \times \left(\frac{1}{green560} - \frac{1}{rededge705}\right)$	[52]
ARIgreen531	$\frac{1}{green531 \times 100} - \frac{1}{rededge705 \times 100}$	[53]
ARIgreen560	$\frac{1}{green560 \times 100} - \frac{1}{rededge705 \times 100}$	[53]
GLI1	$\frac{2 \times green560 - red650 - coastalblue444}{2 \times green560 + red650 + coastalblue444}$	[54]
GLI2	$\frac{2 \times green560 - red650 - blue475}{2 \times green560 + red650 + blue475}$	[54]
GLI3	$\frac{2 \times green560 - red668 - blue475}{2 \times green560 + red668 + blue475}$	[54]
GLI4	$\frac{2 \times green560 - red668 - coastalblue444}{2 \times green560 + red668 + coastalblue444}$	[54]
NDVIred668	$\frac{nearir842 - red668}{nearir842 + red668}$	[55]
NDVIred650	$\frac{nearir842 - red650}{nearir842 + red650}$	[55]
NDREI	$\frac{rededge740 - rededge705}{rededge740 + rededge705}$	[56]
NGRDIred668	$\frac{green560 - red668}{green560 + red668}$	[57]
NGRDIred650	$\frac{green560 - red650}{green560 + red650}$	[57]
NPCI	$\frac{red668 - coastalblue444}{red668 + coastalblue444}$	[58]
R740toR717	$\frac{rededge740}{rededge717}$	[59]

Table A4 The table shows the correlation between the different models applied to the visual scoring done in two different experiments (FIP senescence 2022 and FIP main 2018). See Figure A11 for parameter $t80$ in the experiment of 2022 as example.

	Experiment	parameter	model comparison	correlation value
4	FIP senescence	onsen	cgom_fgom	0.96
10	FIP main 2018	onsen	cgom_fgom	0.94
13	FIP senescence	t80	lin_cgom	0.98
14	FIP senescence	t80	lin_fgom	0.98
15	FIP senescence	t80	lin_pspl	0.99
16	FIP senescence	t80	cgom_fgom	0.99
17	FIP senescence	t80	cgom_pspl	0.99
18	FIP senescence	t80	fgom_pspl	0.99
19	FIP main 2018	t80	lin_cgom	0.98
20	FIP main 2018	t80	lin_fgom	0.99
21	FIP main 2018	t80	lin_pspl	0.99
22	FIP main 2018	t80	cgom_fgom	0.99
23	FIP main 2018	t80	cgom_pspl	0.99
24	FIP main 2018	t80	fgom_pspl	0.99
25	FIP senescence	t50	lin_cgom	0.99
26	FIP senescence	t50	lin_fgom	0.99
27	FIP senescence	t50	lin_pspl	0.99
28	FIP senescence	t50	cgom_fgom	1.00
29	FIP senescence	t50	cgom_pspl	1.00
30	FIP senescence	t50	fgom_pspl	1.00
31	FIP main 2018	t50	lin_cgom	0.99
32	FIP main 2018	t50	lin_fgom	1.00
33	FIP main 2018	t50	lin_pspl	0.99
34	FIP main 2018	t50	cgom_fgom	0.99
35	FIP main 2018	t50	cgom_pspl	1.00
36	FIP main 2018	t50	fgom_pspl	1.00
37	FIP senescence	t20	lin_cgom	0.99
38	FIP senescence	t20	lin_fgom	0.99
39	FIP senescence	t20	lin_pspl	0.99
40	FIP senescence	t20	cgom_fgom	1.00
41	FIP senescence	t20	cgom_pspl	0.99
42	FIP senescence	t20	fgom_pspl	0.99
43	FIP main 2018	t20	lin_cgom	0.98
44	FIP main 2018	t20	lin_fgom	0.97
45	FIP main 2018	t20	lin_pspl	0.98
46	FIP main 2018	t20	cgom_fgom	0.99
47	FIP main 2018	t20	cgom_pspl	0.99
48	FIP main 2018	t20	fgom_pspl	0.99
52	FIP senescence	M	cgom_fgom	0.95
58	FIP main 2018	M	cgom_fgom	0.94
64	FIP senescence	endsen	cgom_fgom	0.93
70	FIP main 2018	endsen	cgom_fgom	0.90
73	FIP senescence	Integral	lin_cgom	1.00
74	FIP senescence	Integral	lin_fgom	1.00
75	FIP senescence	Integral	lin_pspl	1.00
76	FIP senescence	Integral	cgom_fgom	1.00
77	FIP senescence	Integral	cgom_pspl	1.00
78	FIP senescence	Integral	fgom_pspl	1.00
79	FIP main 2018	Integral	lin_cgom	1.00
80	FIP main 2018	Integral	lin_fgom	1.00
81	FIP main 2018	Integral	lin_pspl	1.00
82	FIP main 2018	Integral	cgom_fgom	1.00
83	FIP main 2018	Integral	cgom_pspl	1.00
84	FIP main 2018	Integral	fgom_pspl	1.00

Table A5 Evaluation of the best model using the best RGB (*ExGr*) and SPC (*NDVIred*650) index. The sum of all correlation coefficient values for each model was calculated, furthermore the median as well as the range between the minimal and maximal correlation is shown.

**Model**	**cor sum**		**cor median**		**cor range difference**	
	SPC	RGB	SPC	RGB	SPC	RGB
C-Gompertz	4.849	4.165	0.718	0.678	0.225	0.898
F-Gompertz	4.504	4.427	0.681	0.771	0.454	1.095
P-Splies	2.875	2.002	0.737	0.66	0.229	1.128
linear	2.935	1.959	0.73	0.64	0.136	1.101

Table A6 The table shows the correlation coefficient values and RMSE values calculated between RGB (*ExGR*) and SPC (*NDVIred*650) measurements for the FIP main experiment in the year 2022.

parameter	correlation	RMSE
M	0.729	5.397
onsen	0.658	6.169
endsen	0.536	5.144
t80	0.552	6.76
t50	0.725	5.633
t20	0.667	5.2
Integral	0.718	56.96

A.2 Supplementary Figures

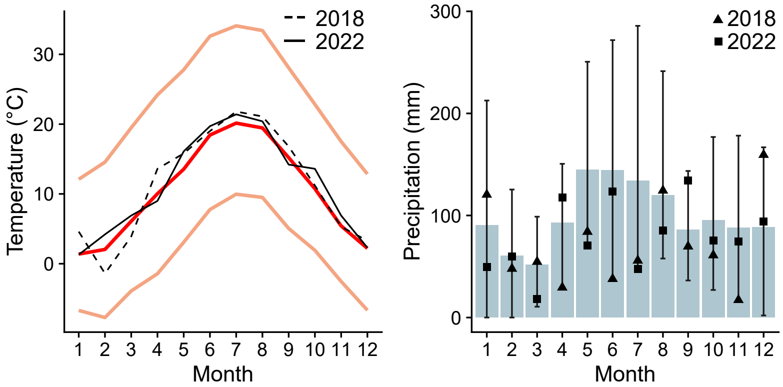

Fig. A1 Left panel shows the monthly mean (dark red line) maximal (upper line) and minimal (lower line) temperature between 2012 and 2022 from the nearby weather station. The black lines show the monthly mean temperature of the year 2018 (dashed) and 2022 (solid) in which the experiments were conducted. The weather station is approximately 500 m away from the field. The right panel shows the monthly mean precipitation in mm. The error bars show the minimal and the maximal measured values within the corresponding month in the period of 2012-2022. The triangles indicate the year 2018, the squares the year 2022 respectively.

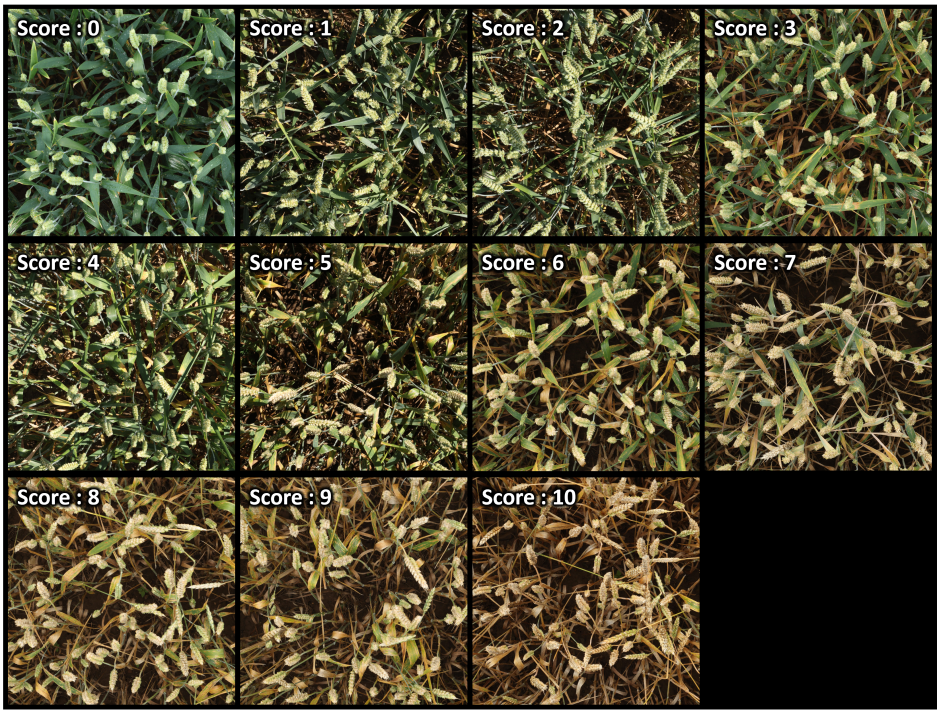

Fig. A2 Images of wheat canopies and corresponding visual senescence scores at the canopy level. Images were acquired at 2.25 m above the ground with a mirrorless digital camera in the 2022 "FIP Senescence experiment". Visual senescence scorings were obtained on the same day directly in the field by inspecting the canopies at a view angle of approximately 45°; images shown here are for reference only and were not used for scoring.

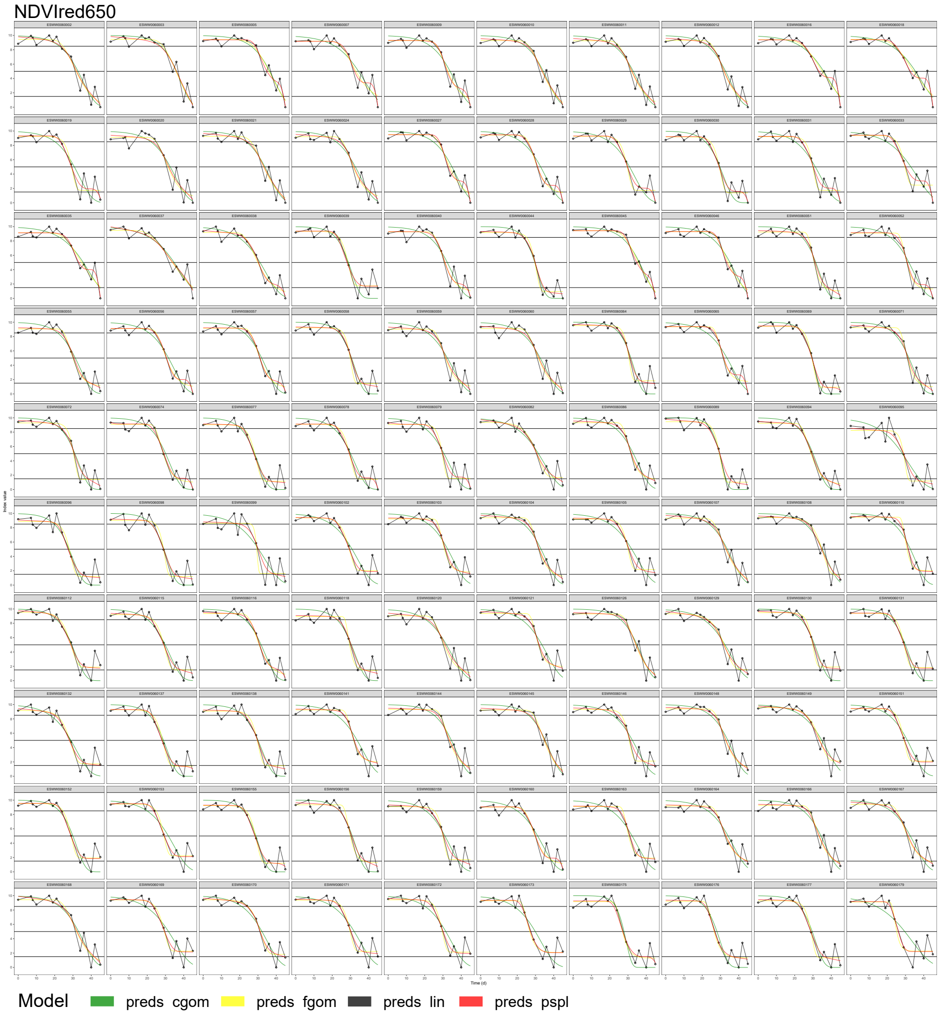

Fig. A3 100 random selected plots (code in the panel header refers to the unique identifier of the plot in the experiment) showing the four different models (lines) as well as the visual scoring (points; y-axis normalized senescence score) over time (x-axis) for the $NDVIred650$ SPC index.

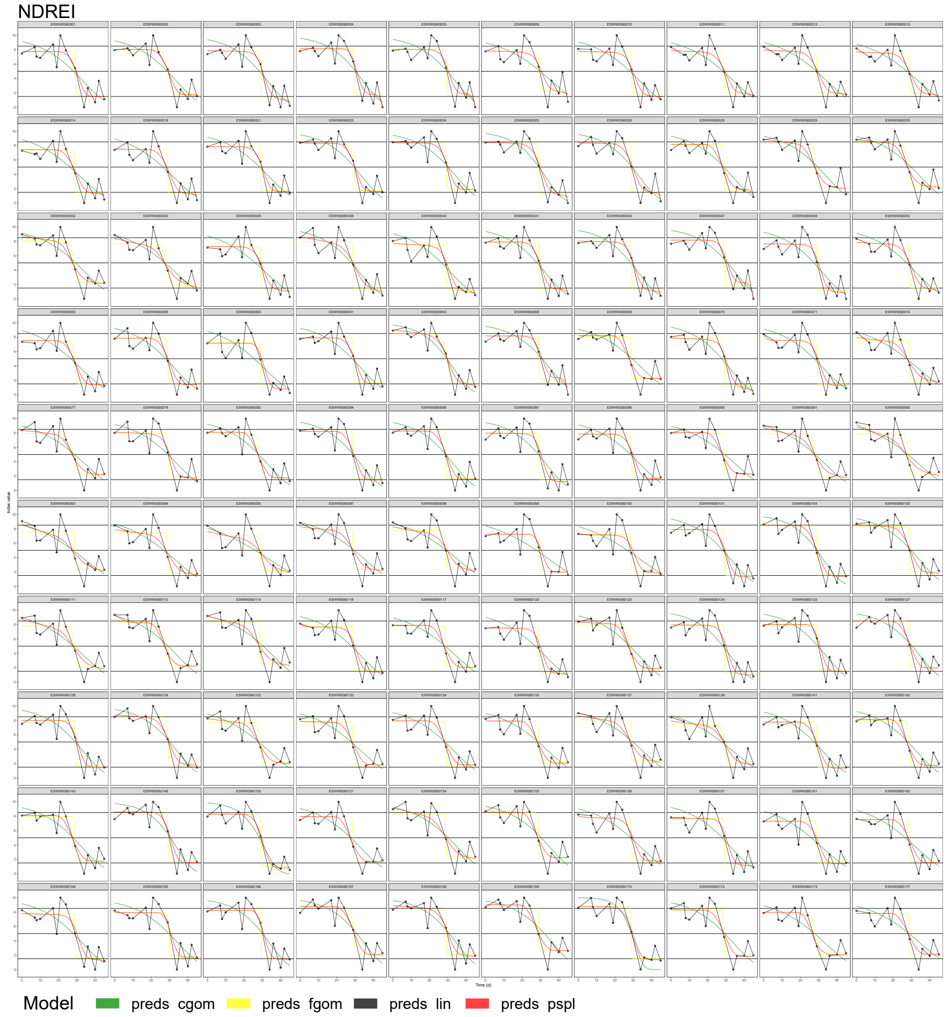

Fig. A4 100 random selected plots (code in the panel header refers to the unique identifier of the plot in the experiment) showing the four different models (lines) as well as the visual scoring (points; y-axis normalized senescence score) over time (x-axis) for the $NDREI$ SPC index.

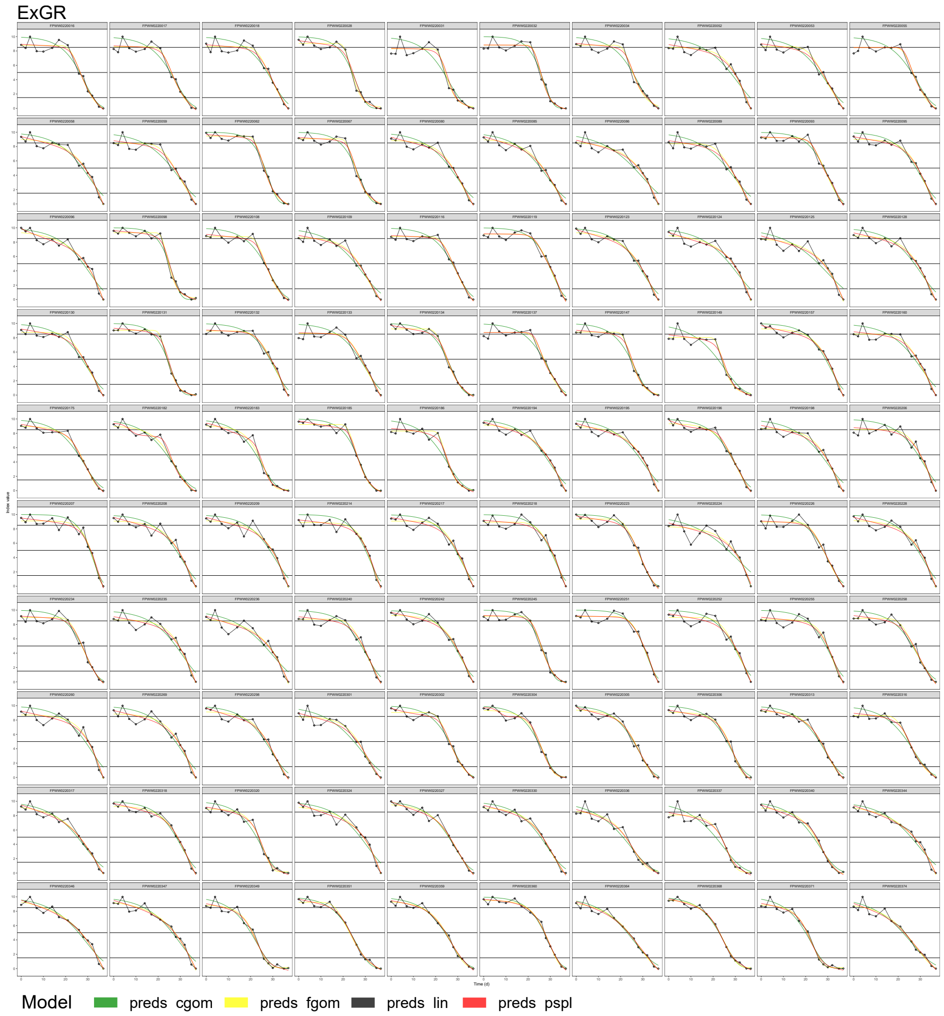

Fig. A5 100 random selected plots (code in the panel header refers to the unique identifier of the plot in the experiment) showing the four different models (lines) as well as the visual scoring (points; y-axis normalized senescence score) over time (x-axis) for the *ExGR* RGB index.

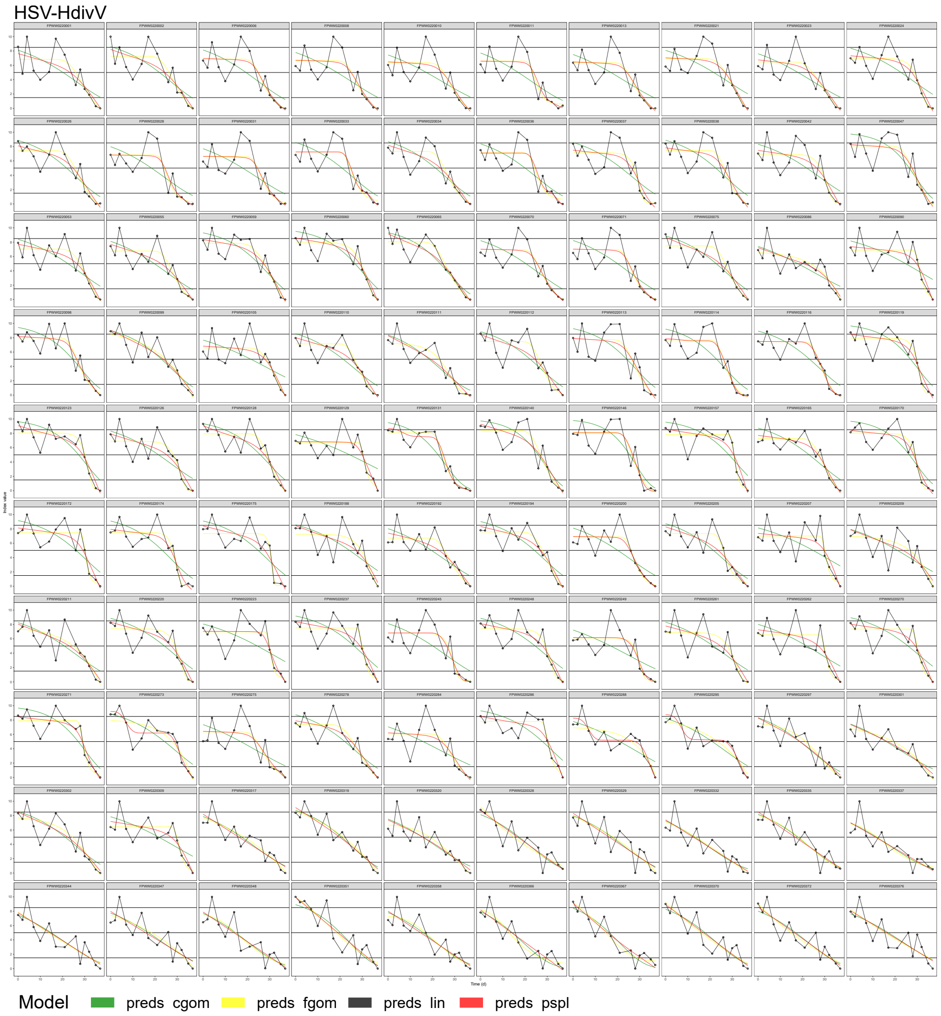

Fig. A6 100 random selected plots (code in the panel header refers to the unique identifier of the plot in the experiment) showing the four different models (lines) as well as the visual scoring (points; y-axis normalized senescence score) over time (x-axis) for the $HSV - HdivV$ RGB index.

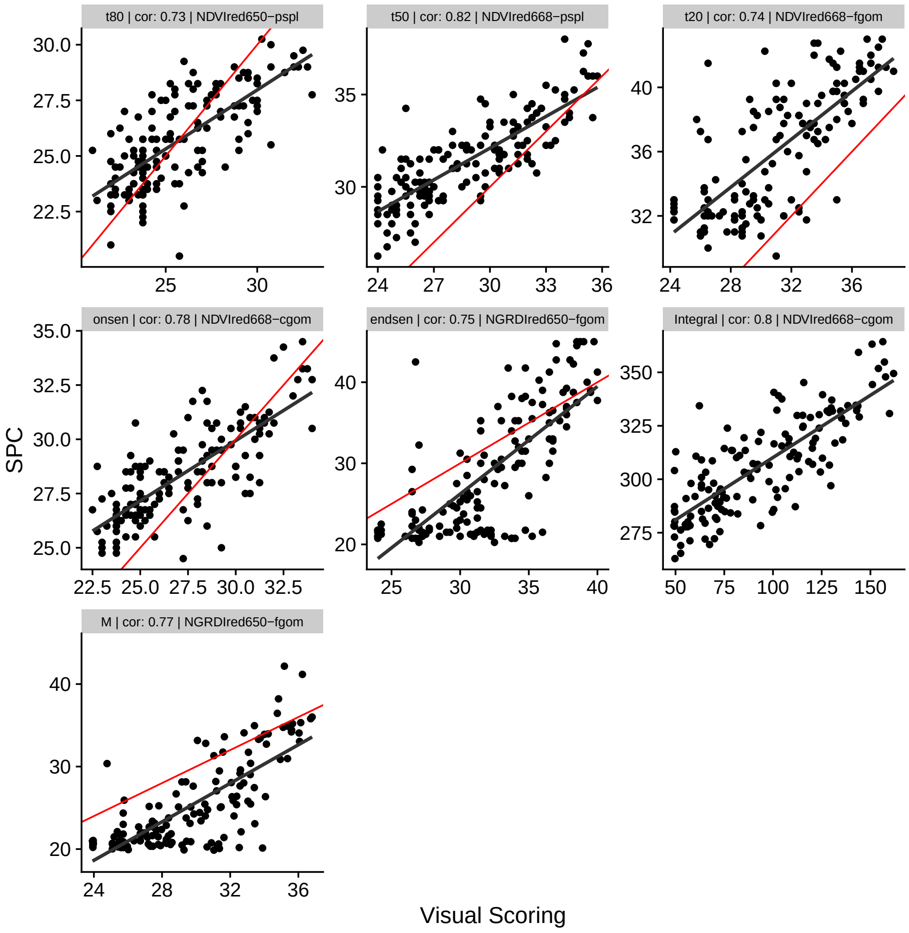

Fig. A7 The Figure shows the correlation between the visual scoring (x-axis) and the best SPC model-index combination per parameter measured by high throughput field phenotyping (HTFP). The red line represent the 1:1 line, whereas the black line shows the correlation fit. Each panel shows one parameter (indicated in the header) and the corresponding Pearson correlation value.

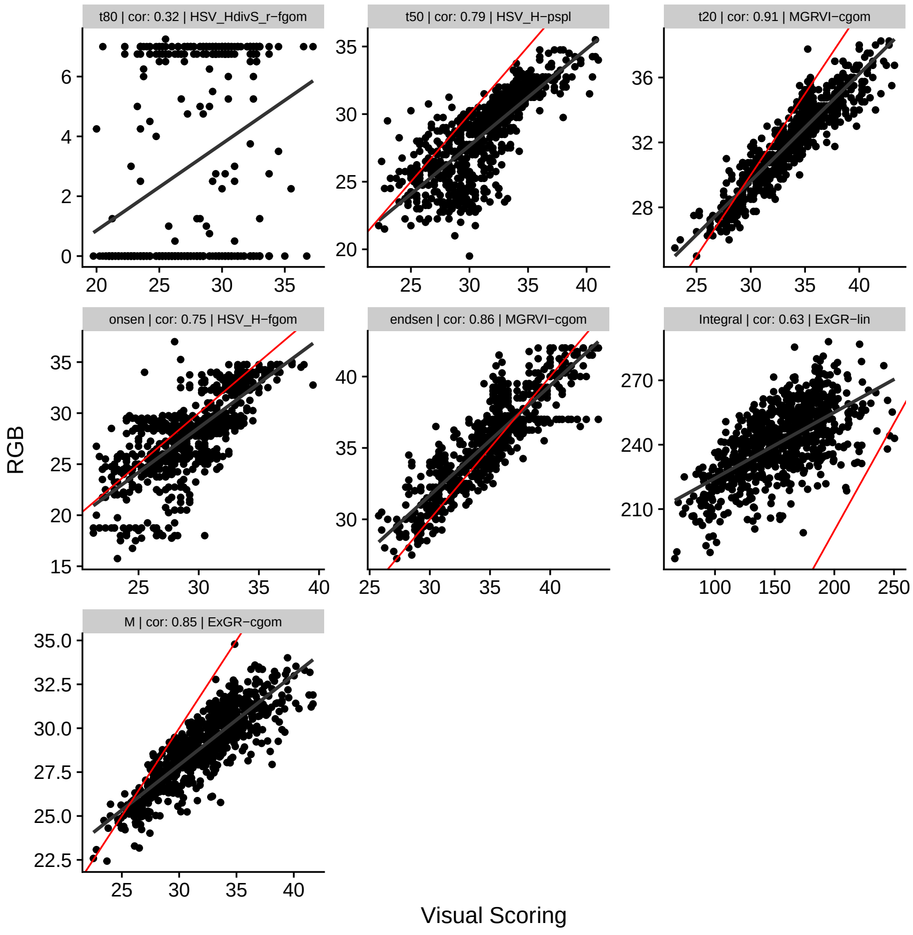

Fig. A8 The Figure shows the correlation between the visual scoring (x-axis) and the the best RGB model-index combination per parameter measured by high throughput field phenotyping (HTFP). The red line represent the 1:1 line, whereas the black line shows the correlation fit. Each panel shows one parameter (indicated in the header) and the corresponding Pearson correlation value.

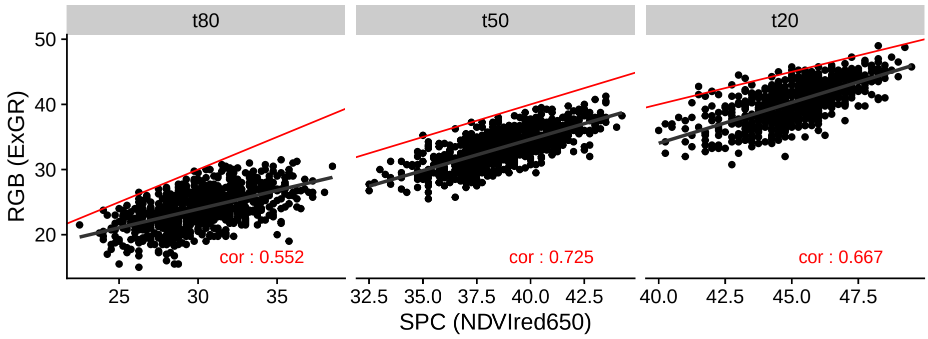

Fig. A9 Correlation between the best RGB index-model combination ($ExGR$ - *cgom* ; x-axis) and the the best SPC index-model combination ($NDVIred650$ - *cgom* ; y-axis). The red line represent the 1:1 line, the black line shows the correlation fit. The columns represent the different parameters ($t20$, $t50$ and $t80$), in each panel the corresponding Pearson correlation value is written in red (see Table A6)

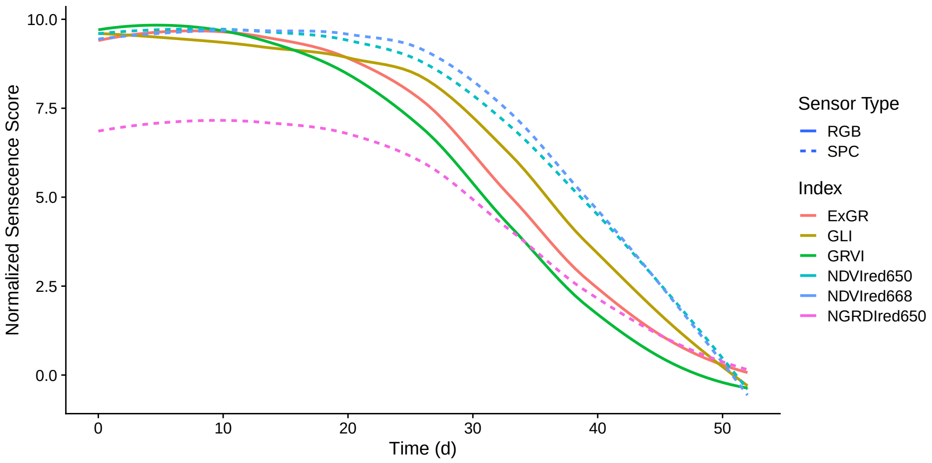

Fig. A10 The figure shows the time (in days) on the x-axis and the normalized senescence score values of different indices on the y-axis. For each of the sensors (SPC in dashed lines and RGB in solid lines), three indices were selected, of which the median values over all plots in the FIP main experiment in the year 2022 were calculated and shown here.

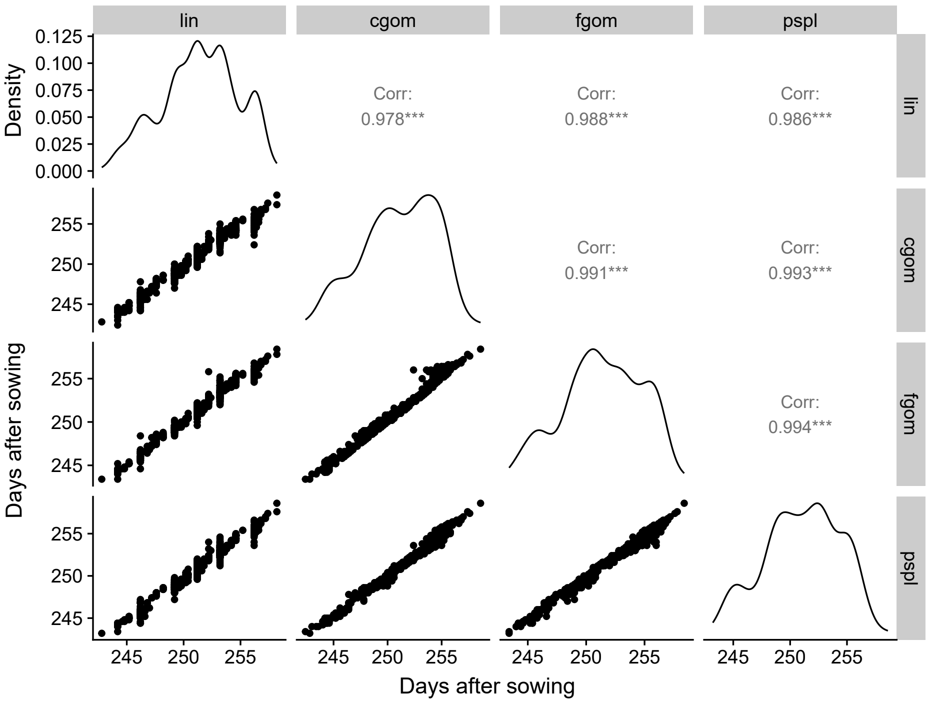

Fig. A11 The figure shows the correlation between the different used models applied to the visual scoring data on both of the experiments in which data was available for the parameter $t80$. Each model was correlated crosswise (lower left) and a correlation coefficient was calculated and shown in the correspond panels (upper right). In the middle line the distribution of the data is shown for each model. Comparable correlations are observed for the other parameters.
